# Epigenetic Modifications of Hormonal Signaling Pathways in Plant Drought Response and Tolerance for Sustainable Food Security

**DOI:** 10.3390/ijms25158229

**Published:** 2024-07-28

**Authors:** Cengiz Kaya, Ferhat Uğurlar, Ioannis-Dimosthenis S. Adamakis

**Affiliations:** 1 Soil Science and Plant Nutrition Department, Harran University, Sanliurfa 63200, Turkey; ckaya@harran.edu.tr (C.K.); fugurlar@harran.edu.tr (F.U.); 2 Section of Botany, Department of Biology, National and Kapodistrian University of Athens, 15784 Athens, Greece

**Keywords:** CRISPR-Cas9, DNA methylation, histone modifications, hormonal signaling, drought stress response

## Abstract

Drought significantly challenges global food security, necessitating a comprehensive understanding of plant molecular responses for effective mitigation strategies. Epigenetic modifications, such as DNA methylation and histone modifications, are key in regulating genes and hormones essential for drought response. While microRNAs (miRNAs) primarily regulate gene expression post-transcriptionally, they can also interact with epigenetic pathways as potential effectors that influence chromatin remodeling. Although the role of miRNAs in epigenetic memory is still being explored, understanding their contribution to drought response requires examining these indirect effects on epigenetic modifications. A key aspect of this exploration is epigenetic memory in drought-adapted plants, offering insights into the transgenerational inheritance of adaptive traits. Understanding the mechanisms that govern the maintenance and erasure of these epigenetic imprints provides nuanced insights into how plants balance stability and flexibility in their epigenomes. A major focus is on the dynamic interaction between hormonal pathways—such as those for abscisic acid (ABA), ethylene, jasmonates, and salicylic acid (SA)—and epigenetic mechanisms. This interplay is crucial for fine-tuning gene expression during drought stress, leading to physiological and morphological adaptations that enhance plant drought resilience. This review also highlights the transformative potential of advanced technologies, such as bisulfite sequencing and CRISPR-Cas9, in providing comprehensive insights into plant responses to water deficit conditions. These technologies pave the way for developing drought-tolerant crops, which is vital for sustainable agriculture.

## 1. Introduction

Climate change presents a formidable challenge to global agriculture, introducing weather variabilities and environmental uncertainties that threaten crop production and food security. Among the most detrimental impacts is drought stress, which compromises crop yield and jeopardizes the sustenance of the growing global population. Addressing this critical issue requires a molecular-level understanding of plant responses to drought stress, which is pivotal for developing drought-resistant crops and ensuring food security [1,2,3]. 

However, plants have to deal with the problem of water shortage in their biochemical and physiological activities. In order to counteract this, plants have developed complex mechanisms that increase their tolerance of stress. Despite significant advances in understanding drought stress, there remains a gap in comprehensively integrating the roles of epigenetic modifications and hormonal signaling across different plant species. These regulatory systems encompass the significant roles of epigenetic modification and hormone signaling in shaping plant perception and responses to drought stress [4,5].

The dynamic epigenetic landscape of plants, characterized by changes in DNA methylation and histone modification over time, is crucial for understanding the mechanisms underlying their response to drought stress [6,7]. These alterations influence chromatin accessibility and structure, ultimately regulating gene expression essential for drought resistance [8]. Plant hormones, particularly ABA, orchestrate sophisticated signaling pathways [9,10], driving various physiological adjustments, such as stomatal closure, improved water use efficiency, and root development. However, existing reviews often focus on either epigenetic mechanisms or hormonal pathways in isolation, failing to provide a comprehensive view of their interplay. Recent studies on the phenomenon of epigenetic memory in drought-adapted plants [11,12] emphasize the pivotal role of epigenetic modifications, encompassing DNA methylation and histone modifications. These modifications are central to governing gene expression and significantly aid in a plant’s resilience to enduring and adapting to harsh environmental circumstances [4,13,14].

In contrast to previous reviews conducted by Yung et al. [15] and Singh et al. [16], our review paper details an in-depth view of the complex molecular mechanisms that plants use to respond to drought stress. Although Yung et al. [15] mainly discuss the epigenetic regulatory mechanisms of abiotic stress responses in legumes and Singh et al. [16] review miRNAs’ functions in post-transcriptional regulation during drought, our study summarizes such findings interactively. We provide an integrated analysis of the combinatorial effects exerted by epigenetic modifications and hormones in controlling plant responses to drought stress. Unlike the targeted focus of Yung et al. [15], our review goes beyond legumes to cover other crops, and hence we offer a wider understanding of drought resilience mechanisms. In addition, unlike Singh et al. [16], who primarily concentrate on the role of miRNAs in drought response, our review discusses the cooperative and interconnected characteristics of hormones and epigenetic changes that positively affect plant fitness against a backdrop of adverse water conditions. By implementing this holistic approach, our review also aims to unravel the molecular intricacies underpinning long-term adaptive strategies, exploring epigenetic memory in drought-adapted plants. Moreover, our exploration extends to the realm of technological breakthroughs, as evidenced in the following sections, to underscore how cutting-edge tools contribute to our evolving understanding of plant behavior under drought stress.

## 2. Epigenetic Memory in Drought-Adapted Plants

Drought stress poses a formidable challenge to global agriculture, necessitating a profound comprehension of plants’ adaptive mechanisms. Beyond immediate physiological responses, recent studies have illuminated the captivating phenomenon of epigenetic memory in drought-adapted plants [11,12]. Epigenetic modifications, encompassing DNA methylation and histone modifications, wield significant influence over gene expression regulation, contributing to a plant’s resilience and adaptation to adverse environmental conditions [4,13,14].

This section explores the emerging field of epigenetic memory in drought-adapted plants, focusing on the transgenerational inheritance of epigenetic changes and the mechanisms involved in the maintenance and erasure of these marks in drought-primed plants. 

### 2.1. Transgenerational Inheritance of Epigenetic Changes in Response to Drought

The phenomenon of transgenerational epigenetic inheritance is a fascinating aspect of plant biology, where stress responses, including those to drought, can be passed down from one generation to the next without changes to the DNA sequence. This means that the offspring of plants that have experienced drought may inherit a predisposition to better withstand such conditions themselves [17,18]. For instance, experiments with *Arabidopsis thaliana* exposed to drought conditions revealed alterations in DNA methylation patterns that persisted in the exposed generation but did not result in transgenerational inheritance of these changes [19]. This indicates that while plants can develop a form of epigenetic memory to aid in their adaptation to drought, this memory does not necessarily persist across generations. Such findings suggest that plants may possess a mechanism allowing them to “remember” previous drought encounters, potentially aiding their adaptation to fluctuating environmental conditions over successive generations.

The molecular basis of transgenerational inheritance is a complex interplay of epigenetic modifications and non-coding RNA molecules that are passed from one generation to the next. This process is crucial for plants, as it allows them to transmit stress responses, such as those to drought, without altering their DNA sequence [18]. For example, in plants, small interfering RNAs (siRNAs) and microRNAs (miRNAs) can induce DNA methylation and chromatin modifications at target loci, leading to transcriptional gene silencing. These siRNAs and miRNAs can be transmitted through the germline to the next generation, maintaining the epigenetic state and the associated phenotype [18]. Additionally, long non-coding RNAs (lncRNAs) have been implicated in transgenerational epigenetic inheritance in plants. LncRNAs can guide chromatin-modifying complexes to specific genomic loci, leading to heritable changes in gene expression [20]. The interplay between non-coding RNAs and classical epigenetic mechanisms, such as DNA methylation and histone modifications, is crucial for the establishment and maintenance of transgenerational epigenetic inheritance in plants [21,22]. 

Histone modifications are another layer of epigenetic regulation involved in the transgenerational inheritance of drought responses. Modifications such as H3K27me3 and H3K4me3 have been implicated in the epigenetic transmission of drought memory. Interestingly, these modifications, which typically have antagonistic roles in the regulation of developmental genes, do not exhibit the same antagonism in genes associated with drought memory. For example, H3K27me3, generally associated with gene repression, may not inhibit transcription in the context of drought. This could be due to the presence of other activating transcription factors induced by drought stress, which can override the repressive effects of H3K27me3 [23].

### 2.2. Maintenance and Erasure of Epigenetic Marks in Drought-Primed Plants

The resilience of plants to drought conditions is significantly enhanced by the transgenerational inheritance of epigenetic changes. However, the mechanisms that maintain and erase these epigenetic marks are just as vital for plant adaptation. A delicate equilibrium is required between the stability of epigenetic memory and adaptability to environmental fluctuations. This balance allows plants to not only survive but also thrive in varying climatic conditions. The dynamic nature of epigenetic regulation in response to environmental stressors is a key factor in the rapid adaptation and survival strategies of plants [12,24].

One of the key mechanisms underlying drought priming is the modification of epigenetic marks. These are chemical changes to DNA and histones that affect gene expression without altering the underlying genetic sequence. In the context of drought stress, epigenetic modifications can lead to the activation or repression of stress-responsive genes, thereby influencing a plant’s ability to cope with water deficit [4]. 

The maintenance of epigenetic marks during drought priming is crucial for the establishment of a “stress memory”. This memory enables plants to respond more rapidly and robustly to subsequent drought episodes. For instance, DNA methylation patterns established during initial drought stress can persist and influence gene expression during later stress events. This epigenetic memory can be maintained for several weeks, allowing the primed plants to retain their enhanced stress tolerance over time [25]. For example, *DRM* and *CMT3* DNA methyltransferase genes are integral to the initiation and maintenance of diverse methylation patterns, essential for the formation of epigenetic memory. Specifically, DRM is involved in de novo methylation, which establishes new methylation marks, while CMT3 is implicated in maintaining methylation patterns during DNA replication and cell division [26]. Histone-modifying enzymes, including histone acetyltransferases (HATs), histone methyltransferases, and histone deacetylases (HDACs), are responsible for regulating the persistence of histone modifications that are indicative of drought stress response [27].

However, the persistence of these epigenetic marks is not indefinite. Over time, or under certain conditions, these marks can be erased, resetting the plant’s stress memory. The erasure of epigenetic marks is a dynamic process that can occur during periods of recovery from stress or during developmental transitions. It ensures that the epigenetic state of the plant remains flexible and responsive to changing environmental conditions [25]. This process is facilitated by enzymes like histone demethylases (HDMs), phosphatases, deubiquitinases, and HDACs, which play a role in the dynamic regulation of histone modifications during periods of drought stress. Histone demethylases, for instance, have been shown to play important roles in plant growth and developmental processes, including responses to environmental cues [28]. Phosphatases contribute to the regulation of abiotic stress signaling and tolerance, as well as plant development [29]. Deubiquitinases are involved in maintaining cellular protein homeostasis and have been implicated in various biological functions within plants [30]. Lastly, histone deacetylases are known to be engaged in how plants react to stress hormones and stimuli, including drought, by regulating the acetylation levels of stress-responsive genes [31,32].

In the model plant *Arabidopsis thaliana*, the SWR1 complex (SWR1C) plays a pivotal role in resetting epigenetic marks, which is integral to the plant’s ability to adapt to environmental stresses such as drought. SWR1C is responsible for replacing canonical histones with variant histones, thereby altering the chromatin structure and influencing gene expression. Mutations in components of SWR1C, including AtSWC6, SUPPRESSOR OF FRIGIDA 3 (SUF3), and PHOTOPERIOD-INDEPENDENT EARLY FLOWERING 1 (PIE1), have been shown to result in developmental defects and altered gene expression patterns, emphasizing the critical nature of epigenetic regulation in plant development and stress response [33]. The SWR1 complex is implicated in a variety of developmental and physiological processes beyond drought stress response, such as DNA damage repair, stress tolerance, and regulation of flowering time. It acts as a key transcriptional regulator that responds to both developmental cues and environmental stresses [34]. The dynamic alteration in the nucleosome structure by the SWR1 complex provides a molecular mechanism for regulating gene silencing, transcription, chromosome segregation, and DNA repair, which are essential for maintaining genetic stability within the cells [35]. 

A study by Forestan et al. [36] investigated the complexity of the epigenetic regulatory network in plants under drought stress, focusing on maize plants facing gentle but long-lasting drought stress before the onset of flowering. Their research identified three categories of stress memory genes: “transcriptional memory” genes with stable transcriptional changes post-recovery, “epigenetic memory candidate” genes with enduring stress-induced chromatin changes without transcriptional alterations, and “delayed memory” genes that store stress signals, leading to a postponed response. 

## 3. Epigenetic Regulation of Hormone Signaling Pathways under Drought Stress

In response to drought stress, plants go through a number of changes that are regulated by hormone signaling pathways. DNA methylation, histone changes, and small RNA-mediated regulation are examples of epigenetic modifications [37,38]. They play critical roles in modifying the hormone signaling pathways [39,40].

**Figure 1:**
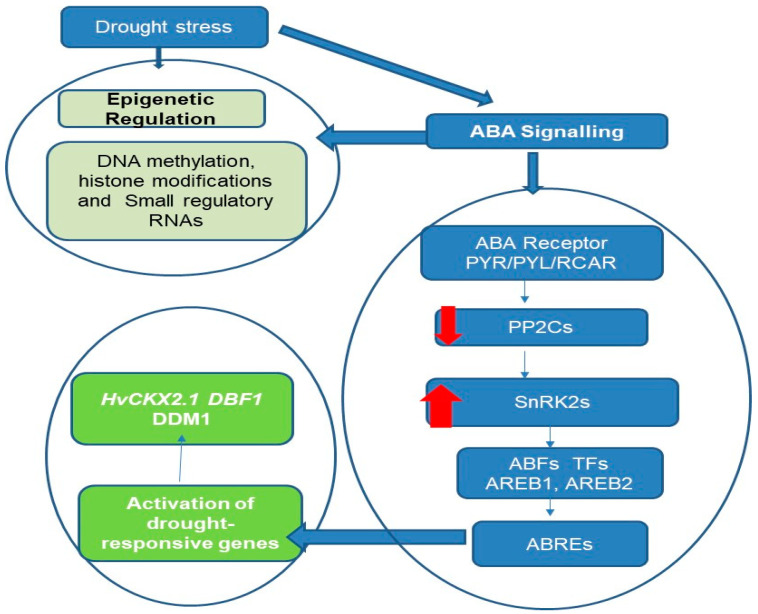
ABA-mediated drought perception and subsequent epigenetic changes in stress response genes. This figure illustrates the crosstalk between ABA-mediated drought perception and subsequent epigenetic changes in plant stress response genes. The left side of the flowchart delineates the epigenetic regulation pathway, initiated by ABA signaling in response to drought stress, leading to key molecular changes such as DNA methylation, histone modifications, and the involvement of small regulatory RNAs. These epigenetic mechanisms contribute to heritable changes that enhance plant resilience to environmental stressors. The right side of the flowchart details the ABA signaling pathway, starting with the ABA receptor PYR/PYL/RCAR. Upon ABA binding, these receptors inhibit the activity of type 2C protein phosphatases (PP2Cs), which are negative regulators of ABA signaling. This inhibition activates Snf1-related protein kinases 2 (SnRK2s), the key positive regulators and secondary messengers in the ABA pathway. The activated SnRK2s phosphorylate transcription factors such as ABFs, AREB1, and AREB2, which bind to ABA-responsive elements (ABREs) in gene promoters. This binding orchestrates the regulation of gene expression, particularly of genes involved in stress responses like *HvCKX2.1*, *DBF1*, and *DDM1*, as well as metabolic pathways, thereby modulating the plant’s response to drought stress. Additionally, some drought-responsive miRNAs contain ABREs in their promoters, and some drought-responsive genes are targets of miRNAs, such as MYB transcription factors, which are repressed through miRNA-mediated mRNA decay to help activate specific responses to drought stress. The red arrows: down for negative regulation, up for positive regulation.

As illustrated in Figure 1, a model of drought-stress-induced epigenetic regulation and ABA signaling pathway crosstalk in stress response genes is proposed.

### 3.1. Influence of DNA Methylation Patterns on Hormonal Signaling Pathways in Plant Adaptation to Drought Stress

The influence of DNA methylation on hormonal signaling pathways during plant adaptation to drought stress is a topic of significant interest within the scientific community [41]. Research on mutants deficient in DNA methylation has illuminated its role in enhancing plant stress tolerance. For instance, Sallam and Moussa [42] investigated the drought stress response of the ABA-deficient maize mutant vp10, observing distinct adaptations compared to wild-type plants. This finding suggests that DNA methylation is crucial in regulating drought stress tolerance and preventing gene expression alterations during stress adaptation. Similarly, Tricker et al. [43] examined the epigenetic response to high vapor pressure deficit (vpd) in *Arabidopsis thaliana*, focusing on the CHROMOMETHYLASE 3 (CMT3) mutant. CMT3 is a plant-specific DNA methyltransferase responsible for maintaining DNA methylation at CHG sites (where H represents A, T, or C). The cmt3 mutant is unable to maintain this asymmetric sequence context of methylation, which significantly impacts stomatal development. This results in a reduced leaf stomatal frequency and improved drought resistance over generations. Symmetric methylation (CG and CHG) is generally stable and heritable, while asymmetric methylation (CHH) is more dynamic and often requires continuous reinforcement. Their study demonstrated that epigenetic modifications in the cmt3 mutant enhanced drought tolerance, underscoring the importance of DNA methylation in plant stress adaptation and resilience.

Epigenetic mechanisms, particularly DNA methylation, play a crucial role in modulating plant responses to drought-induced stress by regulating the expression and activity of genes involved in phytohormone synthesis and signaling [44]. Specifically, DNA methylation is pivotal in enhancing stress resistance through ABA-mediated drought stress tolerance [45]. Studies have shown that DNA methylation patterns undergo significant alterations in response to drought, particularly affecting genes with zinc finger motifs, catabolic enzymes, DNA repair mechanisms, and survival factors [42]. Moreover, research on citrus plants has revealed unique DNA methylation profiles associated with enhanced resilience to water scarcity, indicating an epigenetic basis for improved stress tolerance, particularly through the modulation of abscisic acid (ABA) signaling pathways [45]. However, the use of grafting may not reflect the true heritability of these modifications, as the effects could also be explained by mobile miRNAs transmissible by grafting, such as miR399, miR395, and miR172. Additionally, research suggests that miR156, while not yet confirmed to be mobile, may act as a graft-transmissible signal affecting plant architecture and development in other contexts, as demonstrated in studies with potato [46]. Investigations into barley’s response to terminal drought stress highlighted the role of small regulatory RNAs and RNA-directed DNA methylation in gene silencing under drought conditions, suggesting a potential interplay with hormone regulation pathways. Among the genes affected, CYTOKININ-OXIDASE 2.1 (*HvCKX2.1*) stands out, showing increased DNA methylation at its promoter region under terminal drought stress [47]. In maize, DNA methylation facilitates rapid adaptation to drought stress by modulating gene expression, particularly through the induction of stress-responsive genes via an ABA-dependent pathway. One such pivotal gene is the Dehydration-responsive element-binding factor 1 (*DBF1*), which is activated by drought and salinity through the ABA-dependent pathway in maize seedlings [48]. Furthermore, a study on mulberry leaves revealed substantial alterations in DNA methylation patterns during drought stress, influencing gene regulation across diverse genomic regions and activating vital pathways like plant hormone signaling, particularly auxin. These changes affect the interaction between Aux/IAA proteins and Auxin Response Factors (ARFs), leading to modulation of gene expression in response to drought stress. Differentially methylated genes (DMGs), particularly those associated with spliceosome regulation, phenylpropanoid biosynthesis, carbon metabolism, amino acid biosynthesis, RNA transport, and quorum sensing pathways, play a crucial role in mulberry’s response to drought stress [49]. Furthermore, research on poplar RNAi lines revealed that downregulating the chromatin remodeler DDM1 increased drought tolerance, with significant epigenetic changes observed in genes involved in phytohormone pathways. Specifically, the study found that genes related to phytohormone pathways, including those associated with the downregulation of DECREASED IN DNA METHYLATION 1 (DDM1), exhibited altered methylation patterns, contributing to enhanced drought tolerance in poplar trees. This alteration was accompanied by a physiological shift in hormonal balance, with RNAi-ddm1 lines showing increased salicylic acid and decreased cytokinin levels under drought conditions [50].

In wheat cultivars, one study explored the dynamics of DNA methylation and hormonal changes during drought stress, revealing that the drought-tolerant cultivars showed elevated ABA levels, linked to enhanced drought tolerance. Methylation analysis indicated a reduction in methylation levels during stress, particularly in the CG and CHG contexts, in Bolani, suggesting a role of DNA demethylation in stress response. Furthermore, specific genes affected by DNA methylation changes, such as beta-glucosidase (*BGlu*), phosphoenol pyruvate carboxylase (*PEPC*), glutathione S-transferase (*GST*), glycosyltransferase (*GT*), lysine demethylase (*LSD*), and ubiquitin E2 enzyme genes, were identified. These genes play pivotal roles in the development of drought tolerance [51].

In tea leaves, epigenetic modifications were found to regulate ABA biosynthesis under dehydration stress. This study revealed the upregulated expression of ABA biosynthesis genes in tea during postharvest processing, including ABA-deficient 1 (*CsABA1*), Zeaxanthin epoxidase 2 (*CsZEP2*), 9-cis-epoxycarotenoid dioxygenase 5 (*CsNCED5*), and 9-cis-epoxycarotenoid dioxygenase 3 (*CsNCED3–2*) [52]. Furthermore, studies on self-grafted tomato plants revealed significant epigenetic changes, including DNA methylation, enhancing stress resilience, particularly against drought, indicating the potential involvement of hormone regulation pathways. The analysis identified 630 hypermethylated and 43 hypomethylated differentially methylated genes (DMGs) in H3K4 and 527 hypermethylated and 32 hypomethylated DMGs in H3K27 resulting from grafting [41]. 

Investigation into rice plants highlighted the significant role of DNA methylation in their response to salinity and drought stresses, particularly with implications for gene expression regulation, stress adaptation, and hormone regulation. Specifically, DNA methylation influences how rice plants respond to ABA, indole-3-acetic acid (IAA), gibberellin (GA), jasmonic acid (JA), cytokinins (Cyto), and brassinosteroids (BR), affecting the expression of key stress-responsive genes and contributing to their overall stress resilience [53]. Furthermore, in another study on cotton plants, the significant role of DNA methylation in their response to drought stresses was highlighted, with implications for gene expression regulation, stress adaptation, and hormone regulation. Specifically, this study showed how DNA methylation affects the regulation of key hormones such as ethylene, auxin, gibberellin, and cytokinin, which are critical for managing drought responses and enhancing drought resistance [54]. Similarly, an in silico analysis on rice identified cytochrome P450 (CYP) genes with diverse methylation patterns under various abiotic stresses, suggesting their involvement in stress response mechanisms, including hormone regulation. These genes showed distinct expression profiles in response to treatments with hormones such as ABA, IAA, and GA. Among the identified methylated OsCYPs, notable genes such as LOC_Os09g26950, LOC_Os07g29960, LOC_Os01g43740, and LOC_Os02g17760 displayed varying levels of methylation regulation, potentially implicating them in stress-responsive pathways [55]. In *Populus* × *euramericana*, epigenetic mechanisms, including DNA methylation, influence phenotypic plasticity in response to soil water availability, with coordinated changes in DNA methylation and gene expression observed, particularly in hormone-related genes such as *BRRING-H2* and *MDIS1-IRLK2*. Notably, this study found significant associations between differential gene expression and specific phytohormones. For example, genes related to ABA and SA showed pronounced changes during drought conditions, while ethylene signaling was notably upregulated during rewatering. These findings suggest the role of epigenetic regulation, particularly DNA methylation, in modulating gene expression patterns associated with water stress response and hormone signaling pathways [56]. Lastly, in the orchid *Dendrobium officinale*, DNA methylation dynamics participate in the plant’s response to drought stress, potentially mediated by hormone signaling pathways, including genes such as *DoC5-MTase* and *DodMTase.* These genes contain multiple cis-acting elements, including stress-responsive and hormone-responsive ones. Specifically, hormone-responsive elements were widely observed, including those responsive to ABA (18/70), auxin (9/70), ethylene (6/70), GA (18/70), methyl jasmonate (MeJA) (18/70), and SA (1/70). The presence of these elements suggests that various hormone signaling pathways, particularly ABA, auxin, ethylene, GA, MeJA, and SA, may regulate the expression of these genes, contributing to the plant’s enhanced drought tolerance [44]. These genes have potential functions in polysaccharide accumulation and responses to abiotic stresses, including drought, and they contain multiple cis-acting elements, particularly stress-responsive and hormone-responsive ones, in their promoter regions. This suggests a link between DNA methylation and hormone signaling in response to drought stress. Table 1 summarizes these studies, emphasizing the regulatory function of DNA methylation in hormone-mediated responses to water deficiency.

**Table 1:** DNA methylation-mediated regulation of hormone pathways in drought stress.

Plants	Findings	Mechanistic Focus	Citation
Tomato plants	Self-grafting induces significant epigenetic changes enhancing drought resilience, observed through modifications in histone and DNA methylation patterns, particularly in drought-responsive genes, followed by broader changes in gene expression and hormone regulation.	Epigenetic alterations during grafting and their impact on drought stress response	[41]
Maize	DNA methylation impacts gene regulation and hormone pathways, contributing to plants’ resilience against drought stress.	DNA methylation, hormones	[42]
*Dendrobium officinale*	DNA methylation dynamics, potentially mediated by hormone signaling pathways, contribute to the orchid’s response to drought stress, as evidenced by the differential expression of cytosine-5 DNA methyltransferase (C5-MTase) and DNA demethylase (dMTase) genes.	Role of DNA methylation, including C5-MTase and dMTase genes, in orchids’ adaptation to drought and potential hormone signaling involvement	[44]
Citrus	Divergent DNA methylation profiles in scion/rootstock interactions contribute to enhanced citrus resilience to recurring drought stress.	DNA methylation, ABA	[45]
Barley	Small RNAs, including 24mer hc-siRNAs, are linked to RNA-directed DNA methylation (RdDM) and gene silencing (TGS) under terminal drought stress during grain filling.	RNA silencing, hormones	[47]
Maize	DNA methylation of DBF1 in maize genotypes shows stable upstream methylation under drought conditions, with no specific cytosine methylation patterns in regulatory regions.	DNA methylation, ABA	[48]
Mulberry	Altered DNA methylation patterns in mulberry leaves under drought stress impact hormone signaling and biosynthesis pathways.	DNA methylation, hormones	[49]
Poplar	Downregulation of chromatin remodeler DDM1 in poplar RNAi lines reveals DNA methylation’s role in phytohormone pathways and stress response regulation during water deficit.	DNA methylation, hormones	[50]
Wheat cultivars	Drought-tolerant cultivar “Bolani” exhibits increased abscisic acid (ABA) levels under severe drought conditions, correlating with improved drought tolerance, evidenced by higher radical scavenging activity and maintained relative water content.	Interplay between DNA methylation dynamics, hormonal changes, and drought response	[51]
Tea leaves	Dehydration stress induces epigenetic modifications promoting abscisic acid (ABA) biosynthesis, evidenced by increased expression of ABA biosynthesis genes, the accumulation of ABA, and histone acetylation.	Epigenetic regulation of ABA biosynthesis genes during dehydration stress	[52]
Rice	Zinc finger proteins (ZFPs) respond to salt and osmotic stresses, with tissue-specific expression patterns under stress conditions, revealing epigenetic regulation of gene expression in response to environmental pressures.	Role of ZFPs in epigenetic regulation of gene expression under salt and osmotic stresses	[53]
Cotton plants	Drought stress induces significant DNA methylation changes, including hyper-methylation and asymmetric CHH methylation, potentially regulated by long non-coding RNAs, accompanied by alterations in the methylation of hormone-related genes.	Epigenetic modifications, particularly DNA methylation, and their association with hormone-related genes during drought stress	[54]
Rice	Cytochrome P450 (CYP) genes exhibit diverse methylation levels in response to salinity and drought stresses, with implications for gene expression and hormone regulation, suggesting a role in the plant’s stress response mechanisms.	Influence of CYP genes on rice’s stress response, including their epigenetic regulation and interaction with hormone signaling pathways	[55]
*Populus* × *euramericana*	Phenotypic plasticity in response to soil water availability involves epigenetic mechanisms, with dynamic changes observed in gene expression and DNA methylation, particularly in hormone-related genes, enabling adaptation to water availability.	Coordination between DNA methylation, gene expression, and hormone-related genes in response to drought and rewatering	[56]

**Figure 2:**
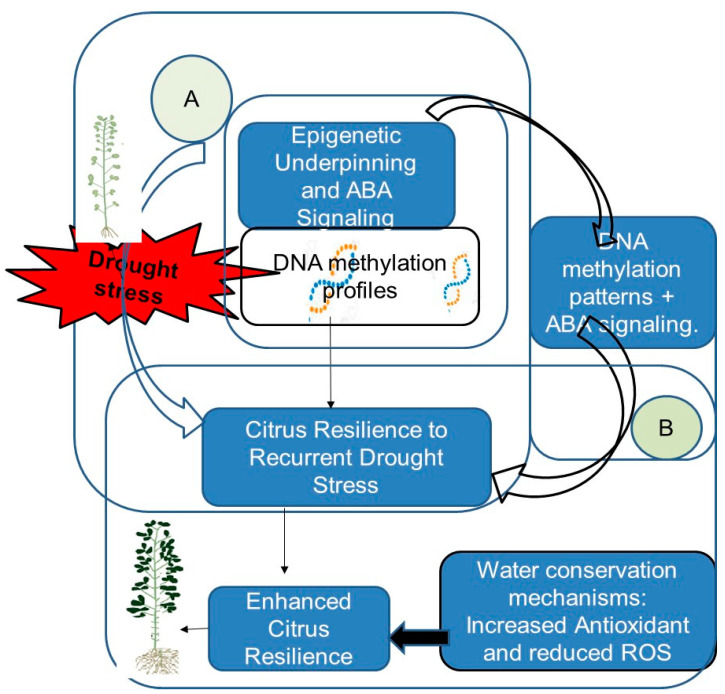
Citrus’ resilience mechanisms to recurrent drought stress: (**A**) Epigenetic Underpinning and ABA Signaling: The observed DNA methylation profiles in scions and rootstocks suggest an epigenetic underpinning. This epigenetic modulation, combined with abscisic acid (ABA) signaling pathways, enhances citrus’ resilience to water scarcity. ABA-mediated mechanisms play a pivotal role in orchestrating adaptive responses. (**B**) Enhanced Citrus Resilience: The interaction between DNA methylation patterns and ABA signaling significantly enhances citrus plants’ ability to cope with recurrent drought stress. This enhanced resilience is evident through physiological responses and antioxidant system activation, both contributing to reduced reactive oxygen species (ROS) production [45].

Figure 2 presents a comprehensive overview of the mechanisms of resilience of citrus to recurrent drought stress.

The gathered studies highlight the essential function of DNA methylation in managing the hormonal signals that plants rely on to adjust to drought conditions. By influencing gene expression and activity, DNA methylation plays a pivotal role in bolstering a plant’s defense against stress. It achieves this by adjusting the production and signaling of phytohormones. Notably, drought-induced changes in DNA methylation patterns have a significant impact on crucial genes that respond to stress across a variety of plants, including maize, Arabidopsis, citrus, barley, and mulberry. Such regulatory mechanisms promote swift adaptation and resilience, underscoring the complex interplay between epigenetic changes and hormonal processes in plants.

### 3.2. The Impact of Histone Modifications on Hormonal Signaling Pathways in Plant Adaptation to Drought Stress

Histone modifications have a critical role in affecting the intricate interplay between hormonal signaling pathways and a plant’s coping strategies for drought stress. Recent research has unveiled the extensive impact of histone modification-driven hormonal regulation on enhancing plant resistance to stress. In tomato plants, self-grafting induces notable epigenetic changes affecting histone and DNA modifications, gene expression, and hormone regulation. One study revealed significant alterations in histone H3 K4 and K27 trimethylation and DNA methylation patterns post-grafting, leading to enduring shifts in gene expression related to hormone levels, chromosomal structure, metabolic processes, and responses to stimuli [41]. Barley’s response to drought conditions involves a complex interplay between epigenetic and transcriptional changes. Global ChIP-seq analysis detected modifications in histone H3, particularly K4 trimethylation and K9 acetylation, induced by drought stress. Notably, H3K9 acetylation’s sensitivity to drought was pronounced, with enrichment observed in genes involved in ABA signaling pathways. Additionally, this study facilitated the discovery of crucial elements in ABA signaling, including the protein phosphatase 2C family (PP2Cs), shedding light on the complex mechanisms of drought response in barley [57]. The pivotal role of *WHIRLY1* in barley’s drought response is highlighted, particularly in modulating stress and developmental pathways. The overexpression of *WHIRLY1* led to changes in ABA marker gene responses and reduced ABA concentrations during drought. Moreover, altered histone modifications in drought-responsive genes suggest a link between *WHIRLY1* expression and the epigenetic regulation of gene activity during drought conditions [58]. 

JmjC domain-containing proteins are crucial in enhancing wheat’s drought tolerance through orchestrating complex epigenetic modifications, particularly histone alterations, closely intertwined with hormonal signaling pathways. Notable genes involved include *Tr-1B-JMJ2*, *Tr-1A-JMJ2*, *Tr-1B-JMJ3*, *Tr-1D-JMJ2*, *Tr-7A-JMJ1*, and *Tr-4B-JMJ1*, among others [59]. The connection between histone deacetylation, hormone signaling, and plant adaptation to drought stress is multifaceted. In tomatoes, the extensively expressed histone deacetylase gene *SlHDA5* is induced by hormones like ABA and methyl jasmonate (MeJA), indicating its role in hormone-mediated stress responses. Histone modifications, particularly the silencing of *SlHDA5*, lead to decreased tolerance to salt and drought stress, highlighting the critical role of histone acetylation and hormone signaling in plant resilience. *SlHDA5* is induced by ABA and methyl jasmonate (MeJA), and its silencing results in increased sensitivity to these hormones, thus impacting plant resilience to salt and drought stresses [60]. 

Chinese cabbage’s adaptation to drought-induced early blooming is influenced by the interplay between histone modification and hormone signaling. The histone H4 protein *BrHIS4.A04* regulates the ABA pathway and photoperiodic flowering genes through histone modification, adjusting hormonal responses to drought stress [61]. Additionally, tomatoes’ adaptation to drought and salt stress is influenced by the complex interaction between histone modifications and hormone signaling, particularly through the histone deacetylase gene *SlHDA3*. The silencing of *SlHDA3* leads to reduced stress tolerance, emphasizing its pivotal role in modulating hormone-related pathways through histone modification. ABA and GA3 influence stress responses, while IAA and SA are crucial for growth and defense mechanisms, with histone modifications affecting their signaling and plant resilience [62]. In sea buckthorn seedlings, histone H3K9 acetylation regulates genes essential for drought resistance, underscoring its importance in hormone-mediated pathways. Specifically, genes related to ABA synthesis and signaling pathways, as well as those involved in flavonoid and carotenoid biosynthesis, are positively regulated by H3K9 acetylation modification in the presence of drought stress. Notable genes include those responsible for ABA synthesis and signaling, including *PP2C2*, *DREB2*, *NAC*, and *SRK2*, which significantly affect the ABA production and distribution pathway, consequently influencing the gene expression in downstream pathways like *ABF2* [63]. 

One study identified a novel RPD3/HDA1-type histone deacetylase, 84KHDA903, in poplar, revealing its role as a supportive regulator in drought stress adaptation. This histone deacetylase exhibited differential expression patterns under drought, salt, and ABA treatments, indicating its involvement in stress-related pathways. The gene *84KHDA903* responded to ABA and drought but not to salt, suggesting its role in modulating ABA signaling pathways related to stress. Transgenic tobacco plants expressing *84KHDA903* showed increased tolerance to drought stress, accompanied by the upregulation of stress-responsive genes such as *NtDREB4*, *NtDREB3*, and *NtLEA5* during the recovery period. This highlights the importance of histone modification in hormone-mediated responses, particularly ABA-mediated responses, to drought stress. This research provides valuable insights into the molecular mechanisms governing plant adaptation to water scarcity [64]. Moreover, histone deacetylation in *Arabidopsis* is significant for orchestrating ABA-mediated drought tolerance, highlighting the regulatory role of histone modifications in plant stress responses. Genes controlled by histone deacetylases HD2A and HD2B are subject to feedback regulation by ABA, which modulates drought tolerance through ABA-induced transcriptional repression. *Arabidopsis* plants with mutations in HD2A and HD2B exhibited improved drought resistance compared to the wild type, which correlated with higher ROS levels, smaller stomatal apertures, and increased expression of drought-resistance-related genes [65].

In summary, histone modifications and hormonal responses are critical for plants to adjust to drought stress. Studies show the significance of hormonal control mediated by histone modification in regulating drought resistance pathways in several plant species. This knowledge highlights the potential for stress-tolerant crop evolution by illuminating the complex network of molecular interactions that enable plants to overcome drought stress difficulties.

Table 2 illustrates the complex interaction between hormone pathways and histone changes in the context of the drought stress response. The table highlights a number of important research works that have uncovered how histone changes influence the complex regulatory network linking hormone signaling, histone modification, and drought resistance in plants.

### 3.3. Epi-miRNAs and Their Role in Drought Stress Response in Plants

Epi-miRNAs, or epigenetically regulated microRNAs, are increasingly recognized for their role in the complex regulatory networks that plants employ to cope with drought stress [66]. These small non-coding RNAs not only regulate gene expression post-transcriptionally but also interact with epigenetic mechanisms, providing a sophisticated layer of control over plant responses to environmental challenges [67]. 

Under drought conditions, plants produce a range of miRNAs that modulate the expression of stress-related genes. For example, a study on *Lycopersicon esculentum* identified several drought-responsive miRNAs, including miR160, miR166, and miR398, which target genes involved in dehydration response and stress tolerance pathways. These miRNAs play critical roles in regulating the expression of transcription factors and proteins that facilitate drought adaptation, such as dehydration-responsive proteins and glycosyltransferases [68]. Furthermore, Chakraborty et al. [66] identified 1,002 miRNAs in various millet species, with 215 miRNAs specifically regulating 155 major drought-responsive genes. These findings highlight the significant role of miRNAs in drought tolerance and suggest that leveraging miRNA-mediated gene regulation could enhance yield and resilience in drought-prone environments.

Moreover, the interplay between miRNAs and epigenetic modifications is crucial for fine-tuning plant responses to various stress, including drought [12]. Epi-miRNAs can influence the expression of DNA methyltransferases and histone-modifying enzymes, thereby affecting the chromatin state and gene accessibility. For instance, miRNAs play a role in drought stress regulation through involvement in ABA biosynthesis and signaling [69]. Most recently, Gao et al. [70] demonstrated the importance of *OsbZIP86* for drought-induced ABA biosynthesis in rice. In the absence of miR2105, it was shown that *OsbZIP86* is upregulated by *OsSAPK10* and enhances the expression of *OsNCED3*, leading to increased ABA synthesis. The generation of miR2105 knockdown and *OsbZIP86* overexpression lines resulted in a higher ABA content, enhanced drought tolerance, lower rates of water loss, and more stomatal closure compared to wild-type plants. Conversely, miR2105 overexpression, *OsbZIP86* downregulation, and *OsbZIP86* knockout lines exhibited reduced ABA levels and diminished drought tolerance.

Despite growing understanding of their significance, the number of experimentally validated epi-miRNAs remains limited. The current research is focused on identifying additional miRNAs that exhibit epigenetic regulation and understanding their functional roles in drought stress response [71].

In conclusion, epi-miRNAs represent a critical component of the regulatory networks that govern plant responses to drought stress. Their ability to integrate post-transcriptional regulation with epigenetic modifications underscores their potential as targets for improving plant resilience to water scarcity. As research progresses, the identification and characterization of these miRNAs will provide valuable insights into their roles in stress adaptation and crop improvement.

### 3.4. Influence of MicroRNA (miRNA) Patterns on Hormonal Signaling Pathways in Plant Adaptation to Drought Stress

MicroRNAs (miRNAs) are small, non-coding RNA molecules crucial for regulating gene expression in plants, particularly in response to various environmental stresses, including drought [72,73]. MiRNAs demonstrate their regulatory expertise by selectively targeting key genes that are crucial for hormone biosynthesis, perception, and signaling, thereby shaping intricate regulatory networks. For example, a complex regulatory network intertwining miRNAs and hormone signaling was uncovered in *Dendrobium huoshanense*’s response to drought stress. This exploration revealed the differential expression of 211 miRNAs, emphasizing miR156, miR157d, and miR160a-5p’s role in modulating auxin and cytokinin responses, thus linking miRNA-mediated hormonal regulation to the plant’s adaptive strategies [74]. In their effort to dissect the drought response in maize roots, researchers underscored the morphological, physiological, and transcriptomic shifts triggered by drought, with implications for gene expression and hormone regulation [75].

Exploring drought-tolerant tomato breeding, one investigation unveiled a network of 699 miRNAs, with miR160, miR165, miR166, miR171, miR398, miR408, miR827, miR9472, miR9476, and miR9552 orchestrating drought stress- and tissue development-related gene modulation through the intricate machinery of hormone signaling [68]. Furthermore, in rice drought stress tolerance, miRNA-mediated hormonal regulation was further unraveled by Chen and Li [76]. Importantly, the functional analysis highlighted enrichment in hormone signaling, metabolism, and antioxidant defense, accentuating the role of these miRNAs in orchestrating *Oryza sativa*’s drought response through the identified web of 13 miRNAs intricately connected with 58 target mRNAs [76].

In pursuit of deciphering miRNA-mediated mechanisms under elevated CO_2_ and drought conditions, a controlled Soil–Plant–Atmosphere Research (SPAR) system was harnessed. The study by Saminathan et al. [77] revealed the intricate regulatory interplay orchestrated by miRNAs, with a focus on TFs, hormone regulators, and carbon metabolism, unveiling the potential for these small molecules to fine-tune crop growth and development under multifaceted stresses in sweet potatoes. Turning the spotlight on drought stress tolerance in *Camellia oleifera*, diverse responses among cultivars were navigated through. Employing mRNA-seq and miRNA-seq, researchers identified differentially expressed genes linked to photosynthesis, chlorophyll metabolism, circadian rhythm, and hormone signaling, elucidating how miRNAs serve as pivotal players in shaping drought tolerance in this species [78]. In an early study, rice’s response to drought was explored using a partial root zone drying (PRD) system. Their comprehensive exploration not only identified novel miRNAs but also highlighted their differential expression under varied moisture regimes. Strikingly, in an intricate interplay, hormonal levels and phosphorous homeostasis emerged as central players in rice’s adaptation to drought stress [79].

Investigating drought stress responses validated the involvement of key miRNAs—miR156, miR159, miR160, miR167, miR171, miR172, miR398, miR403, miR408, miR842, and miR2275—in peach and almond. The study in question highlighted the crucial role of these miRNAs in orchestrating drought adaptation. qPCR analysis confirmed their implication in the dehydration stress response across genotypes. Additionally, predictions from the Rose database unveiled mRNA targets regulated by these miRNAs, including transcription factors. Promoter region analysis identified hormone-responsive elements, indicating potential interactions between miRNAs and hormone signaling pathways in drought stress. Network analysis further elucidated the miRNA–gene target interactions, underscoring the intricate role of miRNAs in drought response [80]. In another early investigation, Deng et al. [81] explored miRNA-mediated hormonal regulation in Paulownia “yuza 1” under drought stress. Their research unveiled a suite of miRNAs influencing plant hormone signaling, photosynthesis, and osmotic adjustment, providing valuable insights into the molecular underpinnings of drought resistance in this species. Specifically, their study detected 107 miRNAs and 42 putative target genes associated with drought stress, with 77 miRNAs showing differential expression between drought-treated Paulownia “yuza 1” and the control. Through functional categorization of the targeted genes, they revealed that Paulownia “yuza 1” could react to drought stress through mechanisms such as plant hormone signal transduction, osmotic adjustment, and photosynthesis.

In the context of rice, Yang et al. [82] further expanded our understanding of miRNA-mediated mechanisms of drought response. Their exploration revealed lncRNAs’ involvement in hormone signaling, chlorophyll synthesis, and protein synthesis pathways, aligning with the finding that lncRNAs are key regulators in plant drought stress responses. Additionally, they elucidated a ceRNA network, shedding light on the intricate genetic mechanisms underlying drought resistance. Specifically, their study identified MSTRG.28732.3 as potentially interacting with miR171 in the chlorophyll biosynthesis pathway, thereby influencing the plant’s drought stress tolerance by modulating key genes such as *Os02g0662700*, *Os02g0663100*, and *Os06g0105350*.

These studies collectively unravel the pivotal role of miRNAs in shaping hormonal signaling pathways, shedding light on how these intricate regulatory networks contribute to plant adaptation to drought stress. MiRNA selectively target genes essential for hormone biosynthesis, perception, and signaling, thereby creating complex regulatory networks that enable plants to modulate their physiological and morphological responses under drought conditions. Through detailed analyses across various species, researchers have highlighted the altered expression of miRNAs and their specific targeted genes. These interactions play crucial roles in hormone signal transduction, photosynthesis, osmotic adjustment, and other metabolic pathways critical for drought tolerance.

The key findings regarding the role of miRNA-mediated hormonal regulation in enhancing drought stress tolerance across various plant species are summarized in Table 3.

## 4. Technological Advances in Studying Epigenetic Modifications

The exploration of epigenetic modifications has become pivotal in understanding the intricate regulatory mechanisms that govern gene expression, especially in the context of plant responses to environmental stresses such as drought. Recent technological advances have revolutionized the study of epigenetic modifications, providing researchers with powerful tools to unravel the complexity of these molecular processes [83]. This section focuses on two key technological breakthroughs: high-throughput techniques for profiling epigenetic changes and the application of CRISPR-Cas9 to engineering epigenetic regulation for drought tolerance.

Table 4 shows the pivotal role of technological breakthroughs in profiling epigenetic changes and engineering epigenetic regulation for drought tolerance.

### 4.1. High-Throughput Techniques for Profiling Epigenetic Changes

The advent of high-throughput technologies has revolutionized chromatin and epigenetic research, allowing for comprehensive profiling of chromatin structures and their genomic accessibility. Techniques such as enzymatic cleavage, transposition, and DNA methyltransferase applications, when coupled with high-throughput sequencing, have provided unprecedented insights into chromatin accessibility [84]. This progress has been pivotal in driving the development of computational epigenetics, expanding our ability to analyze and interpret vast datasets generated by these methods [85].

Bisulfite sequencing stands out as a cornerstone technique for DNA methylation profiling, enabling precise mapping of 5-methylcytosine at single-nucleotide resolution and offering a comprehensive view of the epigenetic landscape within the genome [86]. Studies leveraging these high-throughput methods have illuminated various aspects of plant responses to environmental stressors. For instance, Hua et al. [87] utilized small RNA sequencing to identify the miRNAs involved in drought tolerance in wheat, uncovering both known and novel miRNAs modulated by simulated drought conditions. This led to the discovery of key miRNA targets implicated in critical biological pathways. Similarly, Shaik and Ramakrishna [88] explored a vast array of drought-responsive genes in rice, employing techniques like methylcytosine immunoprecipitation and sequencing to map DNA methylation and investigate miRNA targets. Their findings highlighted a complex interplay between DNA methylation, miRNA activity, and gene expression, particularly in genes associated with chromatin remodeling. It is recommended to use bisulfite sequencing for high-resolution mapping of DNA methylation across different plant cultivars subjected to drought stress. There is a focus on profiling both drought-tolerant and drought-sensitive cultivars to identify differentially methylated regions (DMRs) that correlate with drought resilience, providing insights into key epigenetic markers for breeding programs.

Utilizing high-throughput sequencing (HTS), Zheng et al. [89] established rice lines with accumulated epimutations over multiple generations of drought exposure, demonstrating the non-random nature of epimutation occurrence and its role in enhancing adaptability to drought conditions. Garg et al. [90] employed HTS to investigate the DNA methylation patterns in three rice cultivars with drought stress tolerance, identifying extensive DNA methylation at single-base resolution, with significant differences among cultivars. Differentially methylated regions (DMRs) were linked to the expression of genes crucial to drought stress response, emphasizing the role of DNA methylation in gene regulation under stress.

Chromatin immunoprecipitation sequencing (ChIP-seq) has transformed the study of histone modifications by enabling genome-wide mapping of specific histone marks [91,92]. In recent studies focusing on epigenetic changes under stress conditions, researchers employed ChIP-seq analysis. In the study by Ost et al. [57], this technique identified drought-induced alterations in histone acetylation and trimethylation patterns, revealing their role in shaping the chromatin landscape of barley plants during water deficit conditions. Global ChIP-seq analysis highlighted specific histone modifications associated with ABA signaling and related pathways, leading to the activation of drought-responsive genes. Similarly, Song et al. [93] investigated the H3K9ac levels in *Brachypodium distachyon* under drought stress, revealing variable levels at specific genomic loci and a positive correlation between H3K9ac and gene expression for drought-responsive genes. Dasgupta et al. [94] explored H3K27 modifications in IR64 rice during cold stress, uncovering a correlation between H3K27 modifications and stress-responsive gene activation. Meanwhile, Zong et al. [95] studied the regulatory mechanism of drought-responsive genes in rice, revealing an interplay between histone H3K4me3 modification and the transcription factor OsbZIP23. These studies collectively highlight the utility of ChIP-seq analysis in elucidating the epigenetic mechanisms governing stress responses in plants. Researchers are encouraged to employ ChIP-seq to map histone modifications, such as H3K9ac and H3K27me3, in plants under drought stress. Integrating ChIP-seq data with transcriptomic profiles can elucidate the regulatory roles of specific histone marks in activating or repressing drought-responsive genes. Additionally, targeting histone modifications linked to ABA signaling pathways is recommended for in-depth analysis.

Furthermore, assays like Assay for Transposase-Accessible Chromatin using sequencing (ATAC-seq) provide insights into chromatin accessibility dynamics, providing a global view of the regulatory regions involved in drought response [96]. Mladenov et al. [97] utilized ATAC-seq to reveal the differential chromatin accessibility dynamics in *Haberlea rhodopensis* during desiccation stress, marking the first epigenetic investigation of desiccation-tolerant plants. Yang et al. [98] employed the same technique to illustrate how *OsNMCP1* modifies chromatin accessibility in genes linked to drought resistance in rice, thereby impacting root growth and drought resilience. To advance the research, researchers should utilize ATAC-seq to assess chromatin accessibility and identify the regulatory elements involved in drought response. Conducting comparative studies between different plant species or cultivars can uncover conserved and unique regulatory elements governing drought tolerance, aiding in the identification of candidate genes for genetic engineering.

The integration of high-throughput epigenomic data has facilitated the construction of comprehensive epigenetic maps, contributing to a deeper understanding of the regulatory networks crucial to drought adaptation [83,99,100]. This integration allows for the identification of key epigenetic regulatory elements, unraveling the dynamic modifications occurring within the genome under drought conditions. For example, a study by Wang et al. [101] highlighted the role of DNA methylation in regulating drought tolerance variations in maize roots under water stress. Their comprehensive analysis, which included profiling DNA methylation and the gene expression landscape in maize lines with different drought sensitivities, revealed that increased DNA methylation levels were associated with water stress. Meanwhile, Zhao et al. [102] demonstrated the impact of the histone demethylase Jumonji C domain-containing protein (JMJ710) on the expression of drought-related genes in rice during drought stress, and their study revealed that the overexpression of *JMJ710* led to a drought-sensitive phenotype.

### 4.2. CRISPR-Cas9 Applications in Engineering Epigenetic Regulation for Drought Tolerance

The advent of the CRISPR-Cas9 system has revolutionized genome editing and allowed unprecedented precision in modifying specific DNA sequences [103]. Beyond its conventional use in genome editing, CRISPR-Cas9 has been ingeniously harnessed for epigenome editing, providing a powerful tool for engineering targeted changes in DNA methylation and histone modifications [104,105,106].

CRISPR-Cas9-based DNA methylation editing involves the fusion of catalytic domains, such as the Arabidopsis ROS1 5-meC DNA glycosylase, to a CRISPR-associated null nuclease (dCas9). This fusion enables the precise targeting of DNA methylation-silenced genes for reactivation [107]. This technology has been applied to investigating the causal relationship between DNA methylation patterns and stress responses in plants. By modulating the DNA methylation status of key heterochromatic transposons through knockout mutants of the DECREASE IN DNA METHYLATION1 (*DDM1*) genes in tomato (*Solanum lycopersicum*), researchers have gained insights into the role of *DDM1* in shaping the plant’s ability to maintain epigenetic silencing mechanisms under normal growth conditions [108].

Similarly, CRISPR-Cas9 has been employed for precise histone modification editing. By fusing the Cas9 protein with histone-modifying enzymes, researchers can engineer site-specific changes in histone proteins or modify specific histone marks, allowing for the generation of mutants with altered epigenetic landscapes [109]. This technology has been applied to investigating the causal relationship between histone acetylation patterns and drought tolerance in maize plants. By modulating the histone deacetylase encoded by *ZmHDT103*, researchers have gained insights into the regulatory role of *ZmHDT103* in shaping these plants’ ability to cope with drought conditions. The generation of *ZmHDT103* knockout lines using CRISPR/Cas9 revealed that the mutants exhibited enhanced drought tolerance [110]. Applying CRISPR-Cas9 technology can be recommended for precisely modifying DNA methylation and histone marks associated with drought-responsive genes. This involves targeting key epigenetic regulators like DNA methyltransferases and histone deacetylases to engineer plants with enhanced drought tolerance and validating these modifications by generating and analyzing knockout or overexpression lines.

**Table 4:** Technological breakthroughs in profiling epigenetic changes and engineering epigenetic regulation for drought tolerance.

Techniques	Plant Species	Key Findings	Citations
ChIP-seq technique	Barley	Identification of drought-induced alterations in histone acetylation and trimethylation patterns, revealing the role of epigenetic modifications in barley’s adaptive response to drought	[57]
High-throughput sequencing	Wheat	Identification of drought-responsive miRNAs and targets in drought-tolerant wheat variety XF 20	[87]
High-throughput techniques (HTS)	Rice	Exploration of 5468 drought-responsive genes (DRGs) and their regulatory landscape	[88]
High-throughput sequencing	Rice	Establishment of drought-induced epimutation accumulation lines, highlighting the role of epigenetic mechanisms in rice adaptation to drought	[89]
High-throughput sequencing	Rice	Investigation of DNA methylation patterns and their correlation with gene expression in three rice cultivars with drought stress tolerance	[90]
ChIP-seq analysis	*Brachypodium distachyon*	Exploration of H3K9ac levels in response to simulated drought stress, revealing a positive correlation with gene expression	[93]
ChIP-seq technique	IR64 rice	Investigation of the impact of H3K27 modifications on gene expression during cold stress, highlighting the role of histone modifications in stress-responsive pathways	[94]
ChIP-seq technology	Rice	Exploration of the regulatory mechanism of differentially expressed genes (DEGs) under drought stress, uncovering the interplay between histone H3K4me3 modification and the transcription factor OsbZIP23	[95]
ATAC-seq analysis	*Haberlea rhodopensis*	Investigation of chromatin accessibility dynamics during desiccation stress, providing insights into the epigenetic regulation of desiccation tolerance	[97]
ATAC-seq analysis	Rice	Study on OsNMCP1’s role in altering chromatin accessibility in genes associated with drought resistance and root growth	[98]
ATAC-seq analysis	Maize	Highlighting the role of DNA methylation in regulating drought tolerance variations in maize roots under water stress	[101]
ATAC-seq analysis	Rice	Demonstration of the impact of JMJ710 on the expression of drought-related genes during drought stress, leading to a drought-sensitive phenotype upon overexpression	[102]
CRISPR/Cas9 technology	Maize	Investigation of the causal relationship between histone acetylation patterns and drought tolerance by modulating ZmHDT103, revealing enhanced drought tolerance in knockout lines	[110]

In conclusion, these studies demonstrate the transformative impact of high-throughput technologies and CRISPR-Cas9 applications on our understanding of epigenetic modifications and their role in plant responses to drought stress. Techniques like bisulfite sequencing, ChIP-seq, and ATAC-seq have provided detailed insights into DNA methylation, histone modifications, and chromatin accessibility, revealing complex regulatory networks that govern gene expression under stress conditions. Furthermore, the innovative use of CRISPR-Cas9 for targeted epigenome editing has opened up new avenues for engineering drought tolerance, allowing for precise modifications in DNA methylation and histone marks.

## 5. Future Prospects

Future research should delve deeper into identifying and characterizing specific epigenetic regulators that control DNA methylation and histone modifications in response to drought stress, enabling the development of strategies to modulate these modifications and enhance drought tolerance in crops through techniques like genome editing. Building on our understanding of how epigenetic modifications influence gene expression, scientists could explore using targeted modifications to activate or suppress specific drought-response genes, potentially leading to crops with improved water scarcity resilience. Further investigations should focus on deciphering the intricate crosstalk between hormonal signaling pathways and epigenetic modifications, particularly how hormones like abscisic acid (ABA) and ethylene modulate epigenetic marks, which could reveal new regulatory mechanisms and identify key nodes for manipulation. Additionally, the role of epigenetic modifications in transmitting stress memory across generations warrants exploration to determine how drought-induced changes are inherited and whether they contribute to enhanced resilience, informing breeding strategies that leverage epigenetic inheritance. Exploring environmental epigenetics and epitranscriptomics could uncover how RNA modifications contribute to plant adaptation to drought stress, revealing new layers of regulatory complexity. Adopting integrated omics approaches, combining epigenomics and transcriptomics data, could unravel the intricate molecular networks orchestrating drought response, providing a holistic view of the regulatory landscape. Finally, investigating the epigenetic basis of stress-related traits such as root architecture, stomatal density, and water use efficiency could elucidate how these traits contribute to overall drought resilience.

## 6. Conclusions

In conclusion, epigenetic memory in drought-adapted plants offers fascinating insight into the molecular basis of long-term stress resilience, involving the transgenerational inheritance of adaptive traits. Exploring the maintenance and erasure of these epigenetic marks reveals how plants balance stability and flexibility in their epigenomes. DNA methylation and histone modifications emerge as key mechanisms in plant adaptation to drought, regulating critical genes for drought tolerance and optimizing physiological and morphological responses under water-limiting conditions. Hormonal signaling pathways, including abscisic acid (ABA), ethylene, jasmonates, and salicylic acid (SA) pathways, interact with epigenetic marks to fine-tune gene expression, enabling precise responses to environmental challenges.

High-throughput techniques have unveiled the dynamic nature of DNA methylation, histone modifications, and chromatin accessibility under water deficit conditions. The innovative use of CRISPR-Cas9 to engineer epigenetic regulation has introduced new methods for enhancing drought tolerance in crops. The integration of these technologies promises to decode the complexities of epigenetic regulation and apply this knowledge to develop resilient crops, addressing climate change challenges. The ongoing advancement of these technologies will shape the future of epigenetic research and its applications in agricultural biotechnology.

## Figures and Tables

**Table 2:** Histone modification-mediated regulation of hormone pathways in drought stress.

Plants	Genes	Findings	Mechanistic Focus	Citation
Tomato	Various genes and gene regions	Epigenomic modifications via self-grafting influence drought response and hormone regulation.	Histone modification, hormones	[41]
Barley	Various genes and gene regions	Epigenetic and gene expression changes underlie drought response with ABA involvement.	Histone modification, ABA	[57]
Barley	*WHIRLY1*	Histone modifications affect ABA-related gene expression and drought response.	Histone modification, hormones	[58]
Wheat	*JmjC* genes	JmjC proteins in wheat contribute to drought response and hormone-related pathways.	Histone modification, hormones	[59]
Tomato	*SlHDA5*	Histone deacetylation impacts drought response through hormone signaling.	Histone modification, hormones	[60]
Chinese Cabbage	*BrHIS4.A04*	Histone modification links to hormone signaling in drought-induced flowering adaptation.	Histone modification, hormones	[61]
Tomato	*SlHDA3*	Histone deacetylation affects salt and drought tolerance via hormone responses.	Histone modification, hormones	[62]
Sea Buckthorn Seedlings	Various genes related to ABA signaling	H3K9 acetylation positively regulates drought-related genes and hormone signaling.	Histone modification, hormones	[63]
Poplar	*84KHDA903*	Histone modification aids drought stress adaptation via positive regulation.	Histone modification, drought	[64]
Arabidopsis	*HD2A, HD2B*	Histone deacetylation negatively modulates drought resistance via ABA signaling.	Histone modification, hormones	[65]

**Table 3:** Summary of key findings on microRNA (MiRNA)-mediated hormonal regulation in drought stress tolerance.

Plants	Genes	Key Findings	Citation
Tomato (drought-responsive genotypes)	Drought-related miRNAs	Drought-tolerant tomato breeding involves miRNA-mediated hormonal regulation and stress-related genes.	[68]
*Dendrobium huoshanense*	miRNAs	MiRNAs play a crucial role in Dendrobium’s response to drought, linked to hormone signaling.	[74]
Maize (M8186 variety)	DEMIRs, DEMRs	MiRNAs regulate maize root response to drought, with miR408a impacting reactive oxygen species.	[75]
Rice (Nipponbare cultivar)	miRNAs, target mRNAs	MiRNA-mRNA interactions influence rice’s drought response through hormone signaling and other pathways.	[76]
Sweet potato	Known and novel miRNAs	MiRNAs under elevated CO_2_ and drought conditions target genes related to stress, photosynthesis, etc.	[77]
*Camellia oleifera* Abel.	miRNAs, target genes	Differentially expressed genes related to photosynthesis, hormone signaling, and drought tolerance.	[78]
Rice (IR64 cultivar)	miRNAs	miRNAs regulate rice’s adaptation to drought, targeting transcription factors and hormonal regulators.	[79]
Peach, almond, peach–almond hybrid	miRNAs	Drought-responsive miRNAs impact hormone signaling in peach and almond, aiding drought stress response.	[80]
Paulownia “yuza 1”	miRNAs, target genes	miRNAs and target genes contribute to Paulownia’s drought resistance, impacting hormonal regulation.	[81]
Rice	lncRNAs, miRNAs, mRNAs	lncRNAs, miRNAs, and mRNAs play roles in rice’s drought resistance, with a focus on hormone signaling.	[82]
